# Variations in Clinical Practice: Assessing Clinical Care Processes According to Clinical Guidelines in a National Cohort of Hospice Patients

**DOI:** 10.1177/10499091221100804

**Published:** 2022-05-09

**Authors:** Everlien de Graaf, Matthew Grant, Frederieke van de Baan, Marijke Ausems, Cathelijne Verboeket-Crul, Carlo Leget, Saskia Teunissen

**Affiliations:** 1 Center of Expertise in Palliative Care, Julius Center for Health Sciences and Primary Care, 8124 University Medical Center Utrecht, Utrecht, The Netherlands; 2 8106 The Dutch College of General Practitioners, Palliative Care Physician, Utrecht, The Netherlands; 3 Academic Hospice Demeter, De Bilt, The Netherlands; 4 University of Humanistic Studies, Utrecht, The Netherlands

**Keywords:** hospice, palliative care, delirium, pain, palliative sedation, guidelines, quality care

## Abstract

**Background:** National clinical guidelines have been developed internationally to reduce variations in clinical practices and promote the quality of palliative care. In The Netherlands, there is considerable variability in the organisation and care processes of inpatient palliative care, with three types of hospices – Volunteer-Driven Hospices (VDH), Stand-Alone Hospices (SAH), and nursing home Hospice Units (HU). **Aim:** This study aims to examine clinical practices in palliative care through different hospice types and identify variations in care. **Methods:** Retrospective cohort study utilising clinical documentation review, including patients who received inpatient palliative care at 51 different hospices and died in 2017 or 2018. Care provision for each patient for the management of pain, delirium and palliative sedation were analysed according to the Dutch national guidelines. **Results:** 412 patients were included: 112 patients who received treatment for pain, 53 for delirium, and 116 patients underwent palliative sedation therapy. Care was provided in accordance with guidelines for pain in 32%, 61% and 47% (P = .047), delirium in 29%, 78% and 79% (P = .0016), and palliative sedation in 35%, 63% and 42% (P = .067) of patients who received care in VDHs, SAHs and HUs respectively. When all clinical practices were considered, patient care was conducted according to the guidelines for 33% of patients in VDHs, 65% in SAHs, and 50% in HUs (P < .001). **Conclusions:** The data demonstrate that care practices are not standardised throughout Dutch hospices and exhibit significant variations between type of hospice.

## Introduction

Clinical guidelines have been developed internationally to reduce variations in care provision, enable effective and efficient care, encourage prudence and improve quality of care.^1-4^ There is considerable evidence from a wide range of health settings that variations in clinical practices are associated with poor health outcomes and low-quality care.^5-7^ Clinical guidelines aim to improve structures and processes of care through standardisation of clinical practice, to enable appropriate care for every patient irrespective of their circumstances. As a result, guideline adherence has demonstrated improvements in patient and clinical outcomes, such as improved survival in cancer treatment and symptom management.^2,8,9^

Quality of care is a broad concept that incorporates notions of appropriateness, effectiveness, acceptability, equity, accessibility, efficacy and humanity.^5,10,11^ Patients have described quality palliative care as consistent care; through assessment, communication, clinical decision-making, treatment, and multidisciplinary involvement.^12-14^ Every patient should ideally have access to the same care which is appropriate to their needs, independent of their background, underlying illnesses, and site of care.^13^ The Donabedian model proposes a framework for evaluating health services and quality of care.^8^ According to this framework, health care (and the quality of that care) consists of three domains: structure, process and outcomes. This study measures care processes, as the actions that deliver care, yet these processes reflect care structures and influence outcomes.^15^ As a result, guideline adherence has thus been employed widely as a quality measure associated with all three domains of quality of care.^2^

Dutch national guidelines for palliative care were originally developed in the 1990s, a central impetus being considerable variations in clinical practices evident at the time.^16^ Palliative sedation was a notable clinical practice where there were inconsistencies in care provision identified, and thus concrete guidance was needed to direct best practice care that was evidence-based, collaborative and promoted transparent clinical decision-making.^16^ There are now over forty national palliative care guidelines focusing on differing clinical practices, in order to promote consistent and quality care provision. They consist of evidence-based guidance regarding the aetiology, prevention, diagnosis, management, evaluation and engagement of multidisciplinary care for specific clinical scenarios and populations.^17^

**Figure 1.**
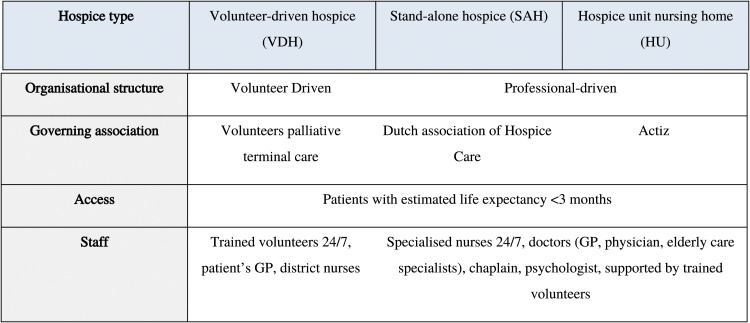
Organisation of hospice care in the Dutch healthcare system^18^,^19^.

The Netherlands contains approximately 300 hospices, which provide inpatient palliative care accessible to patients with an estimated life expectancy of less than three months. They can be divided into three types, including hospices where care is primarily provided by volunteers (VDH), palliative units that operate as part of nursing homes (HU), and independent hospices with specialist-trained palliative care staff (SAH), as described in Figure 1.^18,19^ There are distinct organisations responsible for these types of hospices, which may vary in structures of governance, frameworks of care provision, reporting and quality standards. As a result, hospice care in the Netherlands is subject to considerable structural variability, that may impact upon the processes of care provision, and outcomes.

This study aims to examine clinical practices in palliative care through different hospice types, to identify variations in care processes and settings in The Netherlands. To study these variations in care we focused on three clinical practices with associated national guidelines that have specific processes of care that could be measured through documentation review^20-22^:

• Pain (a symptom),
• Delirium (a syndrome), and
• Palliative Sedation (an intervention).

These practices were identified due to their importance and prevalence in hospice care, as three practices that are fundamental to quality palliative care provision. In particular, palliative sedation is a practice that has received increasing attention in the Netherlands over the preceding decade, in part due to increasing incidence, which in 2017 was reported in 23% of deaths nationwide with higher levels (28%) in the hospice setting.^23-25^ Pain is a very common symptom in hospice populations, the treatment of which is integral to quality palliative care.^22^ Delirium is a syndrome affecting 25-45% of patients admitted to hospices, and for approximately 20% of patients can take an agitated form, that can be very distressing for patients, family members and staff.^21^

## Methods

### Study Design

This retrospective observational cohort study was performed using patient records of patients admitted to hospices in the Netherlands and died in 2017 or 2018. The Strengthening the Reporting of Observational Studies in Epidemiology (STROBE) statement was used for reporting.^26^

### Setting and Participants

Hospices were randomly selected using a random number generator, with equal numbers of VDH, SAH and HUs invited to participate. Hospices for specific patient populations, such as paediatric hospices, were not included. 17 hospices of each hospice type were selected (51 in total), representing geographical locations of the Netherlands.

From each participating hospice an overview was made of all patients who were admitted to the hospice and died in 2017 or 2018. 16 patients from each hospice were randomly selected for data collection. Of these, four patients from each hospice were randomly selected for an in-depth exploration, for which data on clinical practices was collected. Sample size was determine by initial calculations, predicting approximately 200 patients would be required to demonstrate differences in clinical practices between hospice types. It was decided to include all patients (from the 16 selected at each hospice site) that had palliative sedation reported from the mid-point of data collection, not only those who had in-depth analysis.

### Data Collection

Patient clinical records were reviewed by the researchers, who were experienced clinicians (nurses, doctors and allied health) and entered manually using electronic case report forms in Castor Electronic Data Capture System. All data collected was pseudo-anonymised at the level of the patient and hospice. Data collection was developed and tested in an initial pilot in 2017 which has been described elsewhere.^19^ Data was collected by the researchers from December 2018 to June 2021.

Distinct episodes of patient care provision – termed ‘clinical practices’ were identified in the records for these patients, included pain, delirium and palliative sedation. The researchers assessed all available clinical documentation in the first 72 hours after admission, the middle 72 hours, and the last 72 hours, to identify if these clinical practices of pain, delirium, or palliative sedation occurred. Every episode of care related to that clinical practice was recorded, describing the assessment, problem classification, pharmacological and non-pharmacological management, evaluation of treatment and staff member providing care. For pharmacological treatment, type, dosing, and alterations to medication were collected. The clinical care was assessed longitudionally to identify if patient care was escalated appropriately over the individual course of treatment.

**Figure 2.**
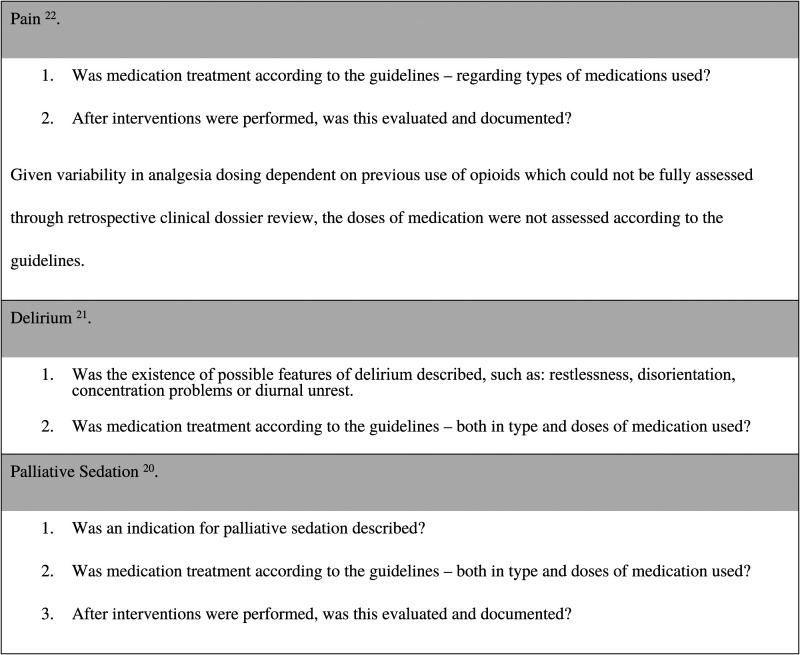
Criteria used to analyse each clinical practice in accordance with national guidelines.

The following criteria described in Figure 2 were assessed for each individual patient in accordance with the national guidelines, which could be reliably measured through the data.

### Ethics

This research was reviewed by the institutional review board of the UMC Utrecht (18-373/C, 18/05/2018) and not considered subject to the Medical Research Involving Human Subjects (WMO) Act of the Netherlands. In line with the principles of Good Clinical Practice, local consent from hospices was obtained.

### Data Analyses

A core team of clinician researchers (EG, ST, MG) analysed the data who had experience as nurses or physicians in palliative medicine. Where there were uncertainties, these were discussed as a group. If the data collected was inconclusive in describing care that did not follow the guidelines, then this was adjudged ‘according to the guidelines’. Data analysis employed SPSS v.26 and comprised primarily of descriptive statistics (proportions, means, and standard deviations where appropriate) and chi-sqaured test for between-group differences.

## Results

192 patients were screened to identify episodes of pain and delirium, and 412 patients screened for palliative sedation. 112 patients (of 192, 58%) were included who received treatment for pain, 53 (of 192, 28%) for delirium, and 116 patients (of 412, 28%) underwent palliative sedation therapy. Mean age was between 72.7 and 75.7 years. Table 1 describes their demographic and clinical characteristics according to hospice type.

**Table 1.** Patient demographics and clinical characteristics.

	VDH	SAH	HU
Pain			
Number patients	34	41	37
Sex–female (%)	23 (68)	20 (49)	19 (51)
Age–mean (range)	75.5 (47-99)	73.4 (51-94)	72.7 (39-91)
Main diagnosis			
-Cancer (%)	26 (76)	36 (88)	30 (81)
-Organ failure (%)	4 (12)	3 (7)	4 (11)
-Neurological (%)	1 (3)	1 (2)	0
-Other (%)	3 (9)	1 (2)	3 (8)
Major comorbidities–mean	.94	.76	1.0
Delirium			
Number patients	21	18	14
Sex–female (%)	11 (52)	5 (28)	5 (36)
Age–mean (range)	79.8 (44-99)	75.4 (56-92)	76.6 (54-90)
Main diagnosis			
-Cancer (%)	13 (62)	15 (83)	8 (57)
-Organ failure (%)	5 (24)	3 (17)	3 (21)
-Neurological (%)	0	0	1 (7)
-Other (%)	3 (14)	0	2 (14)
Major comorbidities–mean	1.23	.83	1.07
Palliative sedation			
Number patients	34	32	50
Sex–female (%)	18 (53)	17 (53)	28 (56)
Age – mean (range)	77.9 (44-99)	71.8 (38-91)	74.6 (42-97)
Main diagnosis			
-Cancer (%)	26 (76)	27 (84)	38 (76)
-Organ failure (%)	2 (6)	2 (6)	5 (10)
-Neurological (%)	1 (3)	2 (6)	1 (2)
-Other (%)	2 (6)	1 (3)	3 (6)
Major comorbidities-mean	1.21	.72	1.22
Days prior to death–palliative sedation initiated			
-Median	1.0	1.0	1.0
-Range	0-7	0-9	0-7

### Pain

The majority (91%) of patients received medication treatment in accordance with guidelines, yet only 49% had routine evaluation after treatment. The proportion of patients for who all criteria were fulfilled was highest in SAH units (61%) and lowest in VDHs (32%). Table 2 describes these results per hospice type.

**Table 2.** Patient treatment for pain in accordance with guidelines (2 criteria) per hospice type.

Type Hospice		VDH	SAH	HU
Medication use according to guidelines	Yes	31 (91.2%)	40 (97.6%)	32 (84.2%)
	No	3 (8.8%)	1 (2.4%)	6 (15.8%)
Evaluation of treatment	Yes	12 (35.3%)	26 (63.4%)	17 (44.7%)
	No	26 (64.7%)	15 (36.6%)	21 (55.3%)
Number of criteria (n = 2) met	0	2 (5.9%)	0	3 (7.9%)
	1	21 (61.8%)	16 (39.0%)	17 (44.7%)
	2	11 (32.4%)	25 (61.0%)	18 (47.4%)

#### Reasons for Medication Treatment Diverging From Guidelines

Ten patients (9%) received medication treatment that did not conform to the guidelines, all of which involved the use of midazolam documented for pain. Three patients were given midazolam as a single agent for pain. Seven patients were treated with midazolam in conjunction with an opioid for pain. None of these patients were documented as receiving palliative sedation at the time of these treatments.

### Delirium

Most patients who experienced a delirium were assessed for anticipation of a prodrome (75%) and had medication treatment in accordance with guidelines (77%). The proportion of patients in which all criteria were met was highest in HU (79%) and SAH (78%) units, and lowest in VDHs (29%). Table 3 describes these results per hospice type.

**Table 3.** Patient treatment according to delirium guidelines (2 criteria) per hospice type.

Type Hospice		VDH	SAH	HU
Anticipation of prodrome	Yes	12 (57.1%)	14 (77.8%)	14 (100%)
	No	9 (42.9%)	3 (16.7%)	0
	Missing data	0	1 (5.6%)	0
Medication treatment according to guidelines	Yes	13 (61.9%)	17 (94.4%)	11 (78.6%)
	No	8 (38.1%)	1 (5.6%)	3 (21.4%)
Number of criteria (n = 2) met	0	1 (4.8%)	0	0
	1	14 (66.7%)	4 (22.2%)	3 (21.4%)
	2	6 (28.6%)	14 (77.8%)	11 (78.6%)

#### Reasons for Treatment Diverging From Guidelines

12 patients received medication treatment that did not conform to the guidelines. Five patients were given benzodiazepines as a single agent and five treated with benzodiazepines prior to anti-psychotics. Two patients were treated with other forms of antipsychotics – quetiapine (step 2 in the guidelines) and dipiperon (not in guidelines) - prior to the use of haloperidol (step 1).

### Palliative Sedation

Patients who underwent palliative sedation therapy had, in most cases, a known clinical indication for therapy documented (90%) and received medication treatment following the guidelines (88%). Treatment was routinely evaluated for 51% of patients. In total, 35% of patients in VDHs met all criteria, 63% in SAHs and 42% in HU units. Table 4 describes these results per hospice type. The four cases of missing data included one case where an indication for palliative sedation was not described and three cases when clinical records regarding medication use did not detail the type of medication used.

**Table 4.** Patient treatment according to palliative sedation guidelines (3 criteria) per hospice type.

Type Hospice		VDH	SAH	HU
Indication for palliative sedation described	Yes	30 (88.2%)	31 (96.9%)	43 (86.0%)
	No	4 (11.8%)	0	7 (14.0%)
	Data missing	—	1 (3.1%)	—
Medication according to guidelines	Yes	28 (82.4%)	28 (87.5%)	46 (92.0%)
	No	5 (14.7%)	3 (9.4%)	3 (6.0%)
	Data missing	1 (2.9%)	1 (3.1%)	1 (2.0%)
Evaluation of treatment	Yes	12 (35.3%)	22 (68.8%)	25 (50.0%)
	No	22 (64.7%)	10 (31.3%)	25 (50.0%)
Number of criteria (n = 3) met	0	2 (5.9%)	0	1 (2.0%)
	1	5 (14.7%)	1 (3.1%)	5 (10%)
	2	15 (44.1%)	11 (34.4%)	23 (46%)
	3	12 (35.3%)	20 (62.5%)	21 (42%)

#### Reasons for Medication Treatment Divergent to Guidelines

11 patients received medication treatment that did not conform to the guidelines. Four patients were given levomepromazine (step 2 in the guidelines) first line for palliative sedation without the use of benzodiazepines (step 1). Two patients were given escalating doses of midazolam at greater than 20 mg/hr without adding levomepromazine (step 2) or specifying a reason why additional sedatives were not considered, both occurring at the same hospice. For five patients, starting doses of midazolam used exceeded specified doses, with four patients having syringe drivers initiated at >2.5 mg/hr (including three patients from the one hospice), or pro ne rata midazolam being initiated at >10 mg for one patient.

### Treatment According to Guidelines for each Clinical Scenario

Treatment was provided conforming to the guidelines for 46% of patients who received treatment for pain, 59% for delirium and 48% for palliative sedation therapy. For each hospice type, patient care was conducted in accordance with the guidelines for 33% of clinical practices in VDHs, 65% in SAHs, and 50% in HUs (P < .001). Table 5 describes the percentage of patients who received care for each clinical scenario according to the guidelines, according to hospice type.

**Table 5.** Percentage of patients for whom all criteria were met for each clinical scenario by hospice type.

	VDH, %	SAH, %	HU, %	P value
Pain	32.4	61.0	47.4	.047
Delirium	28.6	77.8	78.6	.0016
Palliative sedation	35.3	62.5	42.0	.067
All clinical scenarios combined	32.6	64.8	49.5	<.001

## Discussion

### Main Findings of the Study

This study describes how routine palliative care processes were provided to a national sample of Dutch hospice patients for the first time. For approximately 50% of patients, they did not receive care in accordance with national guidelines for that specific clinical practice. Patients in VDH units were less likely to receive care conforming to the guidelines than SAH and HU settings. For patients, this equates to discrepancies in care provision, where the care they receive is influenced by the site of care. These variations are likely due to systemic influences; as can be identified from the discrepancies between types of hospice, and from the reasons for medical care divergent from the guidelines, which identify particular sites where the non-conforming clinical practices are repeated. Such factors may be related to individual hospice policies (or lack of) on specific clinical practices, variations in levels of staff experience and training, and differing approaches to clinical decision-making and documentation.

The study identified that the majority of patients (pain 91%, delirium 77%, palliative sedation 88%) received medication treatment in accordance to the guidelines. The clinical practices that demonstrated most inconsistency in our study were non-medication processes, such as signalling and treatment evaluation, processes that are central to quality care provision.^4^ Hasselaar et al examined palliative sedation practice in The Netherlands between 2003 and 2005 using self-reported questionaires, in which 43% of physicians employed medication treatment that did not follow national guidelines.^27^ This was in contrast to our study, where medication treatment was in most cases, according to the guidelines. It is hoped that this may represent changing clinical practices in The Netherlands as a result of national guidelines first published in 2002.^20^ Whilst the focus of health care professionals is often on practices such as appropriate use of medication, these other processes are equally important, identifying the needs of the patient, guiding treatment, and promoting collaborative care. A retrospective clinical documentation review of six inpatient settings (acute hospitals and palliative care units) from Canada described similar variability in palliative sedation practice.^28^ Indications (85-88%) for palliative sedation and informed consent (73%) were commonly present, yet other practices described in guidelines such as documentation of patient goals of care directed toward terminal management (16%) infrequently occurred.^28^ This study noted substantial variation between care settings, hightlighting how care structures shape processes of care.^28^

### Strengths and Limitations of the Study

The unique contribution of this study is to provide an in-depth ‘snapshot’ of inpatient palliative care practices across the Netherlands throughout different types of hospices. The study has collected extensive data on diverse range of patients throughout The Netherlands, detailing the specifics of their personal and clinical characteristics, care needs, and how and what care was provided, as longitudional data throughout the course of admission. Other studies have used survey methods to examine specific clinical practices from the practitioner perspective, mostly focusing on medication management and referral.^27,29,30^ Whilst these methods are able to access widespread practitioner populations to explore variations in practice, there may be differences between how care is provided by health professionals in theory and in practice. Additionally, these clinical practices are complex processes that are dependent on many individuals. The main strength of this study is that it has enabled an understanding of how patient care occurs in reality across a national sample of hospices, as longitudional care processes involving many individuals.

The methods employed in this study were those deemed most appropriate for the aims of the study, which needed to be able to be replicated in each study site. Whilst these methods enabled such a broad range of variables to be collected, they also confer limitations. Foremost was the reliance upon the quality of clinical documentation to collect data, which in some circumstances were insufficient to fill all variables. There were instances where clinical documentation was sub-optimally recorded, with information missing regarding indications for treatment, non-medication interventions, and exact doses and timing of medications. The interpretations we used to determine ‘care in accordance with the guidelines’ were inclusive, only identifying those care practices that definitely deviated from the guidance, and thus is likely an underestimation of variations in care practices. The outcomes related to these variations in practice differ greatly, as some care processes may have negligible impact on the patient, whilst others, such as very high doses of sedatives, can significantly alter patient care.

The clinical practices described are complex processes, involving many individuals, tasks and influences.^31^ Clinical documentation only describes aspects of these processes, missing many important elements, such as communication, that are key care processes supported by guidelines, yet lacking any definitive outcome. In this study we were only able to examine outcomes that could be measured from the clinical documentation. Many processes were not routinely listed in the clinical notes (i.e., provision of information) in a manner that could be routinely measured through retrospective clinical dossier review. Medication treatment was routinely well documented, and thus forms a central focus of guideline adherence.

### What this Study Adds

The challenge posed by this study is how these variations in care provision can be standardised to promote routine best practice care for each patient. The variations in care observed in this study are substantial and likely to be structural influenced, thus requiring a systematic approach to address these challenges. The logical next step would be consideration of quality measures that could be standardised throughout hospices in The Netherlands.^32^ Through measuring and reflecting on these clinical practices, health care professionals and hospice organisations can identify elements of patient care that can be developed and improved. Further research would ideally focus on identifying care process measures that could be implemented through hospices in The Netherlands, to drive quality improvement and promote evidence-based, consistent care.

Central to all these care processes and their measurement is documentation. Documentation is a fundamental and critical care process that communicates the patient’s needs, responses to care, and care team planning, which has been recognised as integral to quality palliative care.^33-35^ Some care processes, such as medication treatment, may be more readily documented, which reflect the important quality and safety issues related to prescribing.^36^ Hospices act as complex systems where care is provided by a diverse group of regionally involved GP’s, nurses, volunteers and allied health professionals, and thus documentation of all these processes is central to promoting continuity of care.^37^ This is of particular relevance for complex interventions such as palliative sedation, where patient’s needs are often evolving, treatment can have narrow therapeutic windows, and many care providers (both formal and informal) are involved. Care processes should ideally be iterative, transparent and promote continuity between the care team. Developing key quality measures for documentation in palliative care practice may enable hospice services to audit performance, guide hospice teams in which aspects of care should be communicated, and promote its importance as a key driver of care continuity and quality improvement.

## Conclusion

Hospice care in the Netherlands is delivered through many small scale institutions with structural variations in staffing, organisation and reporting. This study describes that there are substantial variations in clinical practices focusing on the treatment of pain, delirium and palliative sedation. When interpreted in relation to the national guidelines, patient care adhered to the guidelines for 65% of cases in SAHs, 50% in HUs and 33% in VDHs. Whilst medication treatment in most cases conformed to the guidelines, there were some institutions where clinical practices that deviated from official guidance were routinely provided. Signalling, evaluation of treatment, and documentation of these practices exhibited greater variability in practice. To improve patient care, care practices need to be further standardised in line with best practice so that each patient is able to access the same quality care.
